# Tapeworm-Induced Eosinophilic Colitis: A Case Report

**DOI:** 10.1155/crgm/8743086

**Published:** 2025-11-07

**Authors:** Mujaheed Suleman, Jay Lodhia, Marianne Gnanamuttupulle, Alex Mremi, Omar Said, Felister Uisso, Ayesiga Herman

**Affiliations:** ^1^Department of General Surgery, Kilimanjaro Christian Medical Centre, P.O. Box 3010, Moshi, Tanzania; ^2^Faculty of Medicine, KCMC University, P.O. Box 2240, Moshi, Tanzania; ^3^Department of Renal & Pancreatic Transplantation, Manchester University NHS Foundation Trust, Manchester, UK; ^4^Department of Pathology, Kilimanjaro Christian Medical Centre, P.O. Box 3010, Moshi, Tanzania; ^5^Kilimanjaro Clinical Research Institute, P.O. Box 2236, Moshi, Tanzania; ^6^Department of Radiology, Kilimanjaro Christian Medical Centre, P.O. Box 3010, Moshi, Tanzania

**Keywords:** albendazole, case report, eosinophilic colitis, intestinal obstruction, Maasai, tapeworm

## Abstract

Eosinophilic colitis is a rare gastrointestinal (GI) pathology characterised by abnormal eosinophilic infiltration into the digestive tract's mucosa. It can present as a primary disorder or as a secondary manifestation, with a wide range of clinical presentations, necessitating a broad differential diagnosis by clinicians. We report an unusual case of eosinophilic colitis caused by a tapeworm in a young male who presented with intestinal obstruction. This case underscores the diagnostic challenges associated with eosinophilic colitis and highlights the essential role of histopathology in confirming the diagnosis. The absence of standardised histological criteria for eosinophil counts in colonic mucosa further complicates the diagnosis. Consequently, management should be individualised, taking into account the patient's condition and the underlying aetiology.

## 1. Introduction

Eosinophilic colitis (EC) is a rare disease of the digestive tract characterised by abnormal infiltration of the colonic mucosa by eosinophilic polynuclear cells [1]. EC is exceptionally rare with only a few cases being reported since 1979. The absence of defined histological criteria for a specific eosinophil count in the colonic mucosa makes the diagnosis of this entity challenging [2]. EC may be primary, without a known aetiology, or secondary to an identified cause. With infiltration of the bowel wall, the ultimate outcome including obstruction and perforation may ensue [3]. In this case report, we present a rare case of EC in a 22-year-old male with large bowel obstruction, with an incidental finding of a long tapeworm as the suspected secondary cause of the colitis.

## 2. Case Presentation

A 22-year-old male from the Maasai tribe presented to our emergency unit with a 1-week history of abdominal distension, bilious vomiting, obstipation and generalised colicky abdominal pain. Prior to this, he had a long-standing history of on-and-off episodes of nocturnal diarrhoea and also reported nonintentional weight loss. The patient had no personal or family medical history, particularly gastrointestinal. He was a pastoralist and had no history of alcohol or tobacco use.

On examination, he was alert and oriented with a Glasgow Coma Scale of 15/15, mildly pale, not jaundiced and not dyspnoeic. His vitals were within the normal range, and he was saturating at 99% on room air. He had a generalised abdominal distension that was tender at the epigastric and umbilical regions, with a normal digital rectal examination. His CBC revealed an elevated leucocyte count of 22.63 × 10^9^/L, predominantly neutrophils at 84%, a normocytic hypochromic anaemia of 10 g/dL and a normal platelet count. The eosinophil count was within the normal range. His serum electrolytes and renal and liver function tests were all normal.

**Figure 1:**
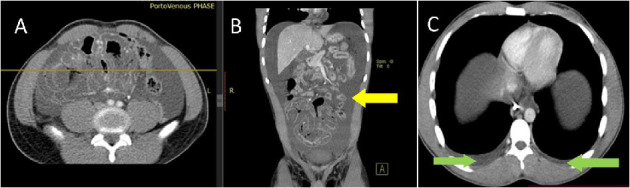
Contrasted abdominal pelvic CT scan. (A) Axial and (B) coronal reformatted showing extensive nonenhancing oedematous mural wall thickening involving the ileal loops with foci of pneumatosis intestinalis and an ulcerative component at mid-distal ileum. Note also gross ascites (yellow), no evidence of mesenteric vessel thrombosis. Mild bilateral pleural effusions (green) more in the right as seen in (C).

**Figure 2:**
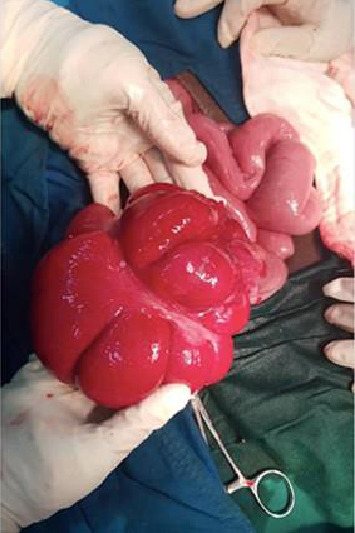
Intraoperative photograph showing an erythematous and distended caecum and ascending colon.

A CT scan of the abdomen revealed extensive mural wall thickening with distended ileal loops and ascites, features were suggestive of small bowel obstruction (Figure 1), and a decision for a surgical exploration was made after counselling and obtaining a written consent. Intraoperatively, a grossly distended hyperaemic caecum and ascending colon, with multiple erythematous patches scattered throughout the colon with mesenteric lymphadenopathy, were found (Figure 2). There was clear amber-coloured ascites of approximately 3 L; however, cytology and biochemistry for the ascites were not done. Other viscera were normal. An ileocaecectomy (10 cm of distal ileum and caecum which had multiple erythematous patches) was done with end ileostomy, and an ascending colon mucous fistula was fashioned. The resected colon segments were taken for histopathology which revealed features suggestive of inflammatory bowel disease. Additional laboratory tests including the ascites fluid were done including Z-stain for tuberculosis which was negative.

**Figure 3:**
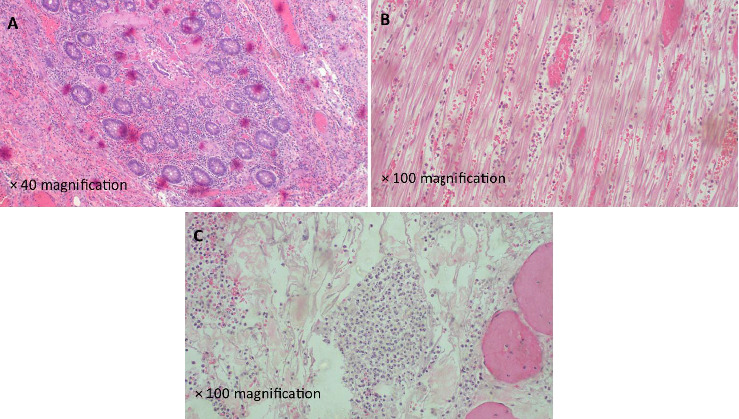
(A) Histopathology of the colon segment demonstrating an inflammatory disorder characterised by prominent eosinophils (H&E staining), (B) photomicroscopy of the colon segment demonstrating marked eosinophilic infiltration in muscularis propria along with other mononuclear cells (H&E staining) and (C) photomicroscopy highlighting a marked oedema and eosinophilic inflammatory infiltrates with eosinophilic abscess formation (H&E staining).

His postoperative recovery was uneventful, with a well-functioning ileostomy and distal mucous stoma. He was discharged with instructions for follow-up at our outpatient coloproctology clinic. At his 2-week review, he was informed of his histopathology results and subsequently referred to medical gastroenterology. There, he was assessed and initiated on sulphasalazine 2 g daily as per hospital IBD management protocol as biologics are not available in a low-resource setting. A follow-up endoscopy of the distal loop stoma revealed areas of colonic mucosal erythema, prompting further biopsies. Histological examination of the second biopsy showed bowel wall oedema, prominent eosinophilic infiltrates and eosinophilic abscesses, consistent with EC (Figure 3).

**Figure 4:**
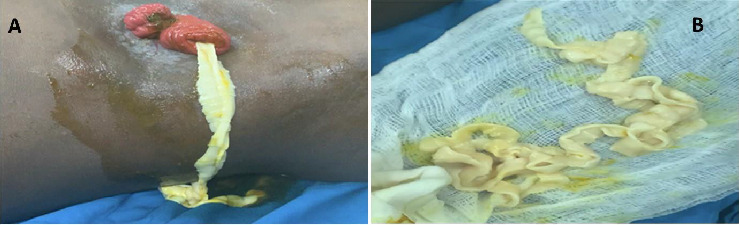
A tapeworm emerging from the ileostomy (A) and part of the removed tapeworm showing proglottids (B).

The patient was prescribed oral albendazole and referred back to the colorectal clinic, where a stoma closure was planned. He was scheduled for stoma closure after 3 months. During the procedure, while preparing the skin intraoperatively, a long tapeworm (*Taenia saginata*) approximately 3 m in length was observed emerging from the ileostomy and was subsequently removed (Figure 4). Stoma reversal surgery, including an ileocolic anastomosis, was performed successfully. Postoperatively, the patient received an additional course of antihelminthic medication (albendazole 400 mg taken orally for 3 days) and was discharged home on the fourth postoperative day. A week later, a stool examination revealed no evidence of parasitic infestation, and the surgical wounds had healed completely. On follow-up 2 months later, he was asymptomatic with no gastrointestinal complaints and was subsequently discharged from our care, with advice to continue routine follow-up at his local general practice.

## 3. Discussion

The first study of eosinophilic gastrointestinal disease (EGID) was published in Germany by Kaijser in 1937. In 1959, the first publication on this pathology in English literature was a case study of EC [4]. In 1970, Klein et al. classified the disease according to the anatomical location of eosinophilic infiltration in the different layers of the intestinal wall into three subtypes: mucosal, muscular and serosal [3].

The prevalence of this disease is hard to estimate, with only 300 cases ever reported from 1937, with an estimate in the United States ranging from 8.4 to 28 cases per 100,000 population [5]. EC can be classified as primary or secondary. Primary EC is related to an allergic reaction, either mediated by IgE and at the origin of an anaphylactic type of food allergy or not mediated by IgE and at the origin of food enteropathy, with milk proteins [5, 6]. On the contrary, secondary EC has identifiable causes including drugs, chronic inflammatory digestive disease, malignant causes, autoimmune damage and hypereosinophilic syndrome and lastly, as in our case, a parasitic infestation [1].

*Taenia* is a tapeworm acquired by ingesting undercooked beef or pork. Taeniasis is more common in Africa, Eastern Europe, Latin America and the Middle East. Most people with taeniasis will have no initial symptoms; however, as time goes on, they will be aware of the infection as they pass proglottids in stool [6]. Several rare complications such as acute obstruction of Wirsung's canal and pancreatitis, gangrenous cholecystitis from an impacted tapeworm in the gallbladder and in the common bile duct, Meckel's diverticulitis, acute abdomen following rupture of a liver abscess caused by the presence of *T. saginata* in the right lobe, acute appendicitis and cholangitis. Intestinal obstruction and perforation of the gut are rare complications considering the worldwide distribution of *T. saginata* infection, with a prevalence rate of up to 10% among some population groups in endemic areas [7].

Bowel obstruction by worms can be caused by either mechanical occlusion or impaction of worms or an inflammatory process leading to bowel wall thickening and eosinophilic infiltrates as seen in our case. Eosinophil-mediated inflammatory responses triggered by helminth infection induce the activation of lymphocyte TH2, overexpression of eosinophil-activating cytokines and generation of antigen-specific IgE antibodies [8]. The physical findings and spectrum of EC range from the level of infiltration of eosinophils in the different layers of the intestinal wall. When the mucosa is infiltrated by eosinophils, patients develop abdominal pain, nausea, vomiting, diarrhoea and malnutrition. Patients with transmural compromises present motility disorders and intestinal obstruction which was seen in our case, and those whose serous layer is compromised may present eosinophilic ascites [7].

The laboratory findings might be indicative of EC, but they are typically not sufficient for a diagnosis. The blood eosinophil count can be normal in up to 20% of patients as in our case. The radiological findings are often nonspecific and only present in 60%–70% of adult cases. CT imaging may show nodularity of the bowel wall, colonic wall thickening, mucosal fold thickening and ascites in some cases. Endoscopic findings are variable and usually nonspecific such as oedematous mucosa with a loss of the normal vascular pattern, patchy erythematous changes, erosions or aphthous ulcerations. Therefore, multiple endoscopic biopsies are required if EC is suspected despite the visualisation of a normal mucosa, as evident in this case presented [5].

Although eosinophils are present in most of the GI tract except the oesophagus, most authors use a cut-off point of 20/HPF. The presence of oedema, eosinophil degranulation, involvement of submucosa and muscularis mucosae and abnormal distribution of eosinophils are useful features for diagnosing EC [9].

Treatment highly relies on the underlying cause, with anti-inflammatory drugs, including steroids; immune-modulatory drugs, including azathioprine and 6-mercaptopurine to reduce the inflammation; antihelminthic drugs to eradicate worm infestation; and lastly, surgery for EC-associated complications including bowel obstruction and perforation [6, 7, 9, 10].

The presented case posed significant diagnostic challenges especially in a low-resource setting. The diagnostic spectrum included inflammatory bowel diseases such as Crohn's disease and intestinal tuberculosis. The incidental finding of the tapeworm at a restorative surgery was an eye opener and calls for surgical and medical gastroenterologists to have EC with its whole spectra of potential causes as part of the differential diagnosis for bowel obstruction.

## Data Availability

The authors have nothing to report.

## Nomenclature

CBC: Complete blood count
CT: Computed tomography
EC: Eosinophilic colitis
GI: Gastrointestinal
HPF: High power field
